# Spatiotemporal Intermittency in Pulsatile Pipe Flow

**DOI:** 10.3390/e23010046

**Published:** 2020-12-30

**Authors:** Daniel Feldmann, Daniel Morón, Marc Avila

**Affiliations:** Center of Applied Space Technology and Microgravity (ZARM), University of Bremen, Am Fallturm 2, 28359 Bremen, Germany; daniel.moron@zarm.uni-bremen.de (D.M.); marc.avila@zarm.uni-bremen.de (M.A.)

**Keywords:** unsteady shear flow, turbulence intermittency, helical instability, puff dynamics

## Abstract

Despite its importance in cardiovascular diseases and engineering applications, turbulence in pulsatile pipe flow remains little comprehended. Important advances have been made in the recent years in understanding the transition to turbulence in such flows, but the question remains of how turbulence behaves once triggered. In this paper, we explore the spatiotemporal intermittency of turbulence in pulsatile pipe flows at fixed Reynolds and Womersley numbers (Re=2400, Wo=8) and different pulsation amplitudes. Direct numerical simulations (DNS) were performed according to two strategies. First, we performed DNS starting from a statistically steady pipe flow. Second, we performed DNS starting from the laminar Sexl–Womersley flow and disturbed with the optimal helical perturbation according to a non-modal stability analysis. Our results show that the optimal perturbation is unable to sustain turbulence after the first pulsation period. Spatiotemporally intermittent turbulence only survives for multiple periods if puffs are triggered. We find that puffs in pulsatile pipe flow do not only take advantage of the self-sustaining lift-up mechanism, but also of the intermittent stability of the mean velocity profile.

## 1. Introduction

**Figure 1:**
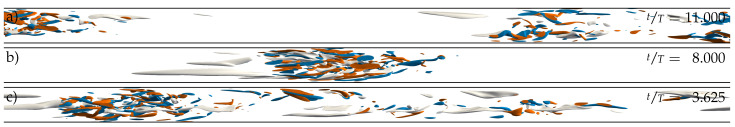
Instantaneous representation of localised turbulent structures in a statistically steady pipe flow (Re=2400, A=0.0). Grey surfaces represent low-speed streaks ($u_{z}^{\prime }=-0.4u_{b}$) and blue/red surfaces represent positive/negative axial vorticity (ωz=±6ubD). (**a**) Puff splitting. (**b**) Single puff. (**c**) Weak slug. The exact location and time for each snapshot are indicated in Figure 2a. The direction of the mean bulk flow ($u_{s}$) is always from left to right.

**Figure 2:**
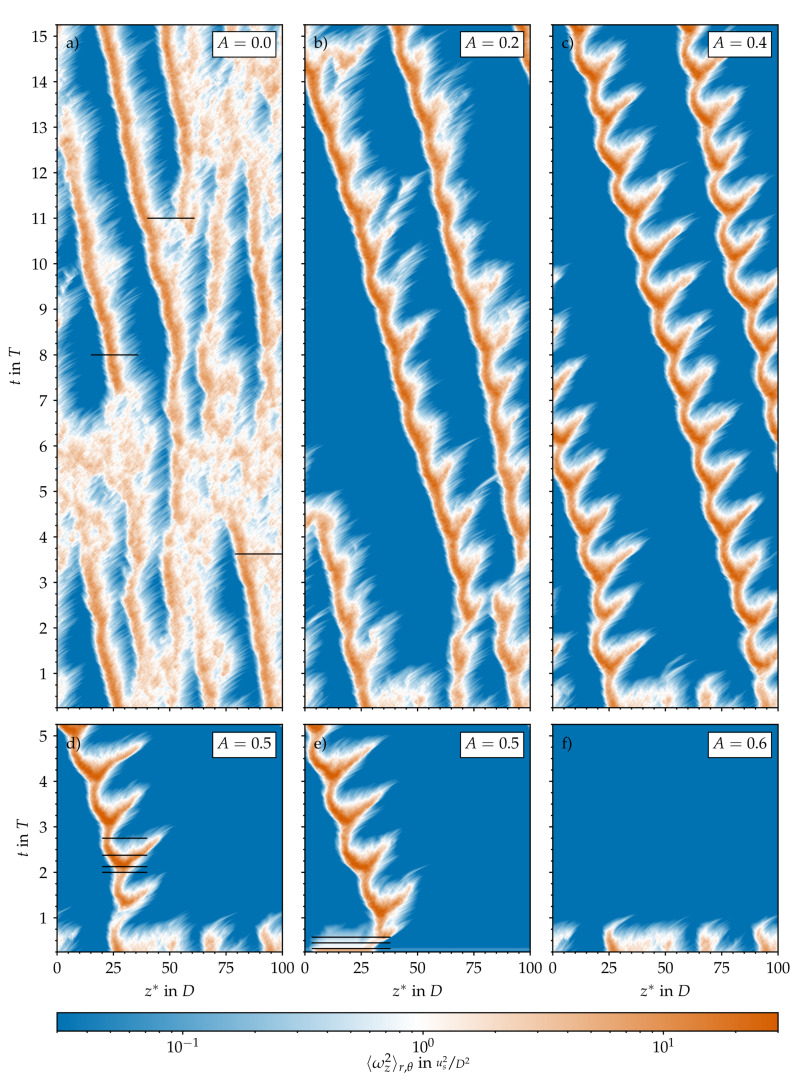
Spatiotemporal representation of the turbulence activity in the computational pipe domain based on the cross-sectional average of the streamwise vorticity ($\omega_{z}$) plotted on a logarithmic scale and in a co-moving reference frame. Steady (A=0, **a**) and pulsatile (**b**–**f**) pipe flow at Re=2400, Wo=8, and different amplitudes *A*. Initial conditions for all A≠0 were either taken from the steady case at time tT=0.25 (**b**–**d**,**f**) or composed of a localised helical perturbation on top of the laminar Sexl–Womersley velocity profile (**e**).

The dynamics and intermittency of transitional turbulence in statistically steady pipe flow have been extensively studied for over a century [1,2,3,4], and the underlying mechanisms are reasonably well understood [5]. The only control parameter is the Reynolds number (Re=ubtDν), which quantifies the relative magnitude of inertia and viscous forces in the system. Here, $u_{b}$, *D*, and ν denote the bulk velocity, the pipe diameter, and the fluid’s kinematic viscosity, respectively. Angled brackets indicate an averaging operation with respect to time (*t*). Although statistically steady pipe flow is linearly stable [6,7], turbulence can be triggered with finite-amplitude perturbations [1,8]. Independently of their type [9], if successful, these perturbations result in spatially localised turbulent puffs, provided that the Reynolds number is not too high [10]. More specifically, for Re<2250, puffs can remain in equilibrium for long times until they either proliferate or decay. Both processes are stochastic (memoryless) and, beyond the critical point (Re>2040), ultimately lead to patterns consisting of several puffs separated by quiescent flow regions [5,11]. For Re>2250, the spatiotemporal dynamics become much richer. Here, puffs may grow and split into two, as for lower Reynolds numbers, or expand continuously to become slugs (see Figure 1). In addition, laminar holes may appear inside the slugs and eventually close, leading to a merger of structures [12]. Figure 2a provides a representation of the resulting spatiotemporally intermittent behaviour of localised turbulent structures (red) and laminar islands (blue) at Re=2400.

In many systems, internal fluid transport is statistically unsteady. Pumps never run perfectly uniformly, blood flow in arteries is pulsatile (due to the systolic contractions of the heart), and air oscillates in and out of the lungs while breathing. A simple mathematical model for these examples is pipe flow driven at a harmonically varying rate

(1) $$\begin{array}{c} u_{b}\left(t\right)={\langle u_{b}\rangle }_{t}\left(1+A\;\cdot \;\cos\left(2\pi \frac{t}{T}\right)\right). \end{array}$$

**Figure 3:**
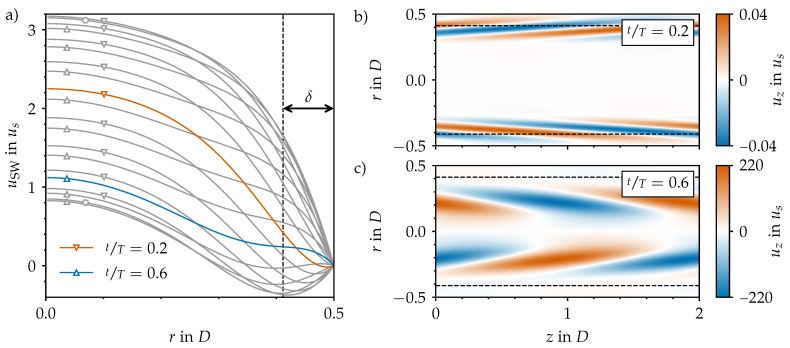
Sexl–Womersley (SW) flow and its optimal perturbation for (Re=2400, Wo=8, A=1.0). (**a**) Time-dependent velocity profile ($u_{\mathrm{SW}}$) for 20 equispaced points within one pulsation period (*T*). Circles denote the maximum and minimum peak flow (PF), whereas upward- and downward-facing triangles denote phases of acceleration (AC) and deceleration (DC), respectively. (**b**) Optimal helical perturbation during DC (tT=0.2) according to our transient growth analysis based on the linearised Navier–Stokes equations. To be used as initial condition in our direct numerical simulation (DNS) (Section 3.3), the helix is scaled to an amplitude of 4 × 10^−2^ $u_{s}$. (**c**) Evolution of the optimal perturbation under the constraints of the linearised Navier–Stokes equations at the later time of maximal energy amplification. Note that, in the framework of transient growth analysis, the absolute amplitude of the initial helix is not important; only the relative growth rate is of interest. The dashed lines correspond to the Stoke layer thickness (δ).

In this case, two more control parameters come in to play in addition to the Reynolds number. The Womersley number (Wo=D22πTν) quantifies the relative magnitude of the viscous time scale with respect to the time scale of the imposed flow pulsation, i.e., the oscillation period *T*. The amplitude (A=uous) is the relative strength of the oscillating component of the flow ($u_{o}$) with respect to the steady component of the flow ($u_{s}={\langle u_{b}\rangle }_{t}$). For A=0, the statistically steady case is recovered, whereas for large *A*, the purely oscillatory flow is approached (as the steady part becomes negligible). According to Sexl [13] and Womersley [14], there is an analytical solution to the Navier–Stokes equations for laminar flow through a smooth pipe and single harmonic driving. The Sexl–Womersley (SW) velocity profile ($u_{SW}\left(r,t\right)$) can be added to the (parabolic) Hagen–Poiseuille profile to obtain an analytical (laminar) solution for any combination of Wo and *A*. As an example, we show in Figure 3a the temporal evolution of $u_{SW}$ for a pulsatile pipe flow at Wo=8 and A=1.

Understanding the transition to turbulence in statistically unsteady pipe flows remains incomplete, although progress has recently been made [15,16,17]. The puff dynamics for relatively small amplitudes (A≤0.5) are well understood. For Wo≤5, the flow stays for a long time in the low Reynolds number regime. A low instantaneous Re enhances the decay of puffs, and hence, puffs only survive if the mean Reynolds number is substantially increased with respect to the steady case [15,16]. For Wo≥12, the minimum Reynolds number necessary for puffs to survive tends asymptotically to the one for statistically steady pipe flow [15,16,18,19]. For intermediate Womersley numbers, the threshold decreases smoothly from the low to high Wo regime [15,16]. This can be seen, for example, in Figure 8 of Xu et al. [15].

Puffs, however, are not the only mechanism through which pulsatile pipe flow may become turbulent. A new instability was discovered recently in laboratory experiments by Xu et al. [17]. In their experiments, curvature, misalignment of pipe segments, small contractions, and, in general, finite-size geometric imperfections led to the cyclic development of sudden bursts of turbulence. At each period, helical-like structures grew and triggered turbulence during the deceleration phase of the pulsation before the flow relaminarised again during the acceleration phase. This behaviour was observed for relatively high amplitudes (A≥0.5), intermediate Womersley numbers (5≤Wo≤8), and mean Reynolds numbers as low as Re=800. Motivated by this finding, Xu et al. [20] carried out a comprehensive non-modal stability analysis of pulsatile pipe flow. They showed that certain helical perturbations exploit an Orr-like mechanism to grow by several orders of magnitude in energy. They linked this mechanism to the inflection points of the SW velocity profile that emerge during the deceleration phase (see Figure 3a–c). Inflectional SW velocity profiles are indeed known to be linearly unstable in the quasi-steady limit [21], as long as they satisfy the Fjortoft criteria. The smaller the Womersley number, the longer the velocity profile is unstable, thus effectively providing a more fertile ground for instabilities to grow. However, as the Womersley number is reduced, the velocity profile becomes increasingly parabolic and, hence, loses its inflection points. The amplification of helical disturbances is most efficient for Wo≈7 [20], exactly in the regime where the helical instability was observed experimentally [17].

The purpose of this paper is to investigate the spatiotemporal intermittency of turbulence in pulsatile pipe flow. More specifically, we aim to characterise the intermediate regime in which helical structures and puffs are expected to compete. To that end, we perform transient growth analysis and direct numerical simulations of pulsatile pipe flow at fixed Re=2400 and Wo=8, as well as different pulsation amplitudes *A*.

## 2. Numerical Methodology

### 2.1. Governing Equations

We consider a viscous fluid with constant properties confined in a straight smooth rigid pipe of circular cross-section and diameter *D*. The fluid flow is driven through the pipe with a time-dependent pressure gradient, and is considered to be incompressible and governed by the Navier–Stokes equations (NSE)

(2) $$\begin{array}{c} \frac{\partial u}{\partial t}+\left(u\;\cdot \;\nabla \right)u=-\nabla p+\frac{1}{Re}\nabla^{2}u+F_{d}\left(t\right)+F_{p}\left(r,\theta ,z,t\right)\;\mathrm{and}\;\nabla \;\cdot \;u=0. \end{array}$$

Here, u and *p* denote the fluid velocity and pressure. The driving force $F_{d}\left(t\right)$ represents a mean pressure gradient, which is adapted in a way such that the flow rate ($u_{b}$) given in Equation (1) is maintained. The additional body force term $F_{p}\left(r,\theta ,z,t\right)$ is used to model geometric imperfections in the pipe geometry, and thus to perturb the flow locally (see Section 2.4). Unless otherwise stated, all quantities are rendered dimensionless using the pipe diameter *D*, the statistically steady part of the bulk velocity $u_{s}={\langle u_{b}\rangle }_{t}$ (see Equation (1)), and the fluid’s density (ρ).

### 2.2. Direct Numerical Simulation

In order to study the departure from the laminar Sexl–Womersley flow and the dynamics of intermittent localised turbulence, we perform direct numerical simulations (DNS) using our open-source pseudo-spectral simulation code **nsPipe** [22]. In **nsPipe**, the governing Equation (2) are treated in cylindrical coordinates $\left(r,\theta ,z\right)$ and discretised using a Fourier–Galerkin ansatz in θ and *z* and high-order finite differences in *r*. No-slip boundary conditions are imposed at the solid pipe wall and periodic boundary conditions in θ and *z*. The discretised NSEs are integrated forward in time using a second-order predictor–corrector method with variable time-step size; details are given in López et al. [22] and the references therein. We have modified **nsPipe** to account for a time-dependent driving force that maintains a pulsating flow rate according to Equation (1) and an additional volume force $F_{p}$ to perturb the flow locally.

We use a computational domain of 100D in length, and the number of radial grid points and Fourier modes used in our DNS is $\left(N_{r}\times N_{\theta }\times N_{z}\right)=\left(96\times 192\times 2400\right)$. After dealiasing, this results in a spatial resolution of $\Delta \theta R^{+}=3.1$ and $\Delta z^{+}=3.8$, whereas radial grid points are clustered towards the pipe wall such that $0.06\leq \Delta r^{+}\leq 1.4$, and 14 points lie within the buffer layer based on the shear Reynolds number for the statistically steady case (A=0). By comparison to the resolution used in other contemporary DNS studies in the literature and the fact that we consider only moderate *A* (the instantaneous Reynolds number is never >(1+A)Re), we expect our choice of resolution to be sufficient for all set-ups considered here. The adaptive time-step size is roughly Δt=2×10−3Dus.

### 2.3. Transient Growth Analysis

In order to study the linear stability of the SW profile and to determine the perturbations that grow the most on top of it within one pulsation period, we have performed transient growth analysis (TGA) for the parameter space at hand. This non-modal method returns the most dangerous perturbation in terms of energy growth out of all possible axial/azimuthal wavenumbers and pairs of initial ($t_{0}$) and final ($t_{f}$) times. To this end, the governing Equations (2) are linearised (LNSE). The LNSE and their adjoint counterpart are integrated forward and backward in time iteratively, until such an optimum is reached for each combination of Re, Wo, and *A*. During integration, the underlaying velocity profile develops in time (see, e.g., Figure 3a), but remains unchanged by the developing perturbations.

The LNSE and their adjoint are discretised using a Fourier–Galerkin ansatz in θ and *z* and a Chebyshev collocation method in *r*. Further details are given in Barkley et al. [23], and our TGA computations were undertaken using an in-house Matlab script.

### 2.4. Modelling Geometric Imperfections in Our DNS

Section 3.6 presents results from DNS in which we mimiced the geometric perturbation of the experiments of Xu et al. [17]. Inspired by the optimal baffle designed by Marensi et al. [24], we here model the effect of geometric perturbations with an additional volume force in Equation (2) of the form

(3) $$\begin{array}{c} F_{p}\;\left(r,\theta ,z,t\right)=-A_{p}\;\cdot \;f_{p}\;\left(r,\theta ,z\right)\;\cdot \;u\left(r,\theta ,z,t\right). \end{array}$$

The body force $F_{p}$ acts against the velocity field u and is localised in the radial, azimuthal, and axial direction by

(4) $$\begin{array}{cc} f_{p}\;\left(r,\theta ,z\right) & =f\;\left(r\right)\;\cdot \;g\;\left(\theta ,z\right)\;\cdot \;h\;\left(z\right)\;\mathrm{with} \end{array}$$

(5) $$\begin{array}{cc} f\;\left(r\right) & =\frac{1}{2}+\frac{1}{\pi }\arctan\;\left(M_{r}\left(r-r_{0}\right)\right), \end{array}$$

(6) $$\begin{array}{cc} g\;\left(\theta ,z\right) & =\frac{1}{\pi }\left(\arctan\;\left(M_{\theta }\left(\theta -\pi \left(\theta_{0}\left(z\right)-L_{\theta }\right)\right)\right)-\arctan\;\left(M_{\theta }\left(\theta -\pi \left(\theta_{0}\left(z\right)+L_{\theta }\right)\right)\right)\right), \end{array}$$

(7) hz=1πarctanMzz−z0+Lz2−arctanMzz−z0−Lz2and

(8) $$\begin{array}{cc} \theta_{0}\;\left(z\right) & =1+2\Delta \theta \frac{\left(z-z_{0}\right)}{L_{z}}. \end{array}$$

These localisation functions satisfy the constraints $\max\left(f\right)=1$, $\min\left(f\right)=0$, $\max\left(g\right)=1$, $\min\left(g\right)=0$, $\max\left(h\right)=1$, and $\min\left(h\right)=0$; the perturbation amplitude is given by $A_{p}$.

**Figure 4:**
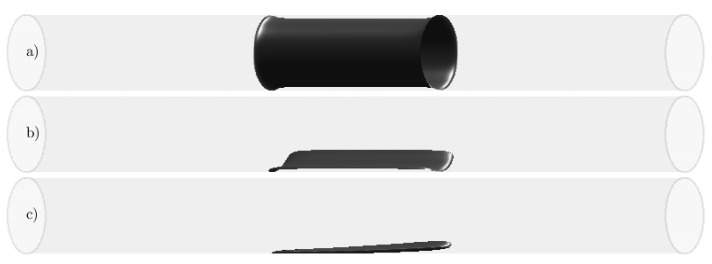
Geometric representation of the perturbation force ($F_{p}$) in terms of iso-surfaces (black) of the localisation function for $f_{p}=0.5$. (**a**) Axisymmetric contraction. (**b**) Localised bump. (**c**) Tilted bump. See Table 1 for details. The direction of the mean bulk flow ($u_{s}$) is always from left to right.

Due to the big parametric space in hand, we designed three simple body force set-ups and left further optimisation of parameters as future work. The first set-up is an axisymmetric force that models the effect of a small circumferential contraction similar to weak stenosis in blood vessels [25] or imperfect pipe joints in laboratory experiments [17] (see Figure 4a). The second set-up is a highly localised force that approximates the effect of a single bump or an individual roughness element (see Figure 4b). The third set-up is also a highly localised force that approximates the effect of a single bump or an individual roughness element, but this time, it is tilted with respect to the axial direction (see Figure 4c). The parameters defining the perturbations are given in Table 1. We studied the effect of the axisymmetric force on steady laminar Hagen–Poiseuille flow at Re=2400 to select a suitable value of the force amplitude $A_{p}$. Our criterion was that the force must be strong enough to sufficiently disturb the flow without creating too long of a re-circulation region. For $A_{p}=0.25$, we found a fair compromise between these two constraints.

The goal of this model is to serve as a proof of concept. Our hypothesis is that geometric imperfections employed in the experiments locally modify the flow pattern causing the instability. The model satisfies this requirement, as it represents a small perturbation to the flow. It is meant for testing such a hypothesis, whereas the precise shape of its geometry plays an ancillary role. In order to faithfully reproduce the experiments of Xu et al. [17], one would need to have a boundary-fitted mesh or use immersed boundary methods. We are, however, confident that if the DNS was exactly reproducing the precise imperfections of the experiments, the exact same behaviour would be observed in the DNS.

## 3. Results

**Figure 5:**
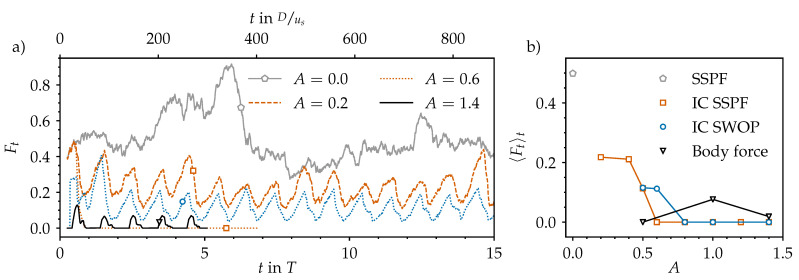
Turbulent fraction ($F_{t}$) in the computational pipe domain based on the axial vorticity data shown in Figure 2 and Figure 7. The threshold to distinguish turbulent from laminar regions is set to ${\langle \omega_{z}^{2}\rangle }_{r,\theta }=4\times 10^{-2}$. (**a**) Time series of the turbulent fraction for several amplitudes *A* (line styles) and different numerical set-ups (symbols and colours from those in (**b**)). (**b**) Time-averaged turbulent fraction ${\langle F_{t}\rangle }_{t>2}$ for four different set-ups: The statistically steady pipe flow (SSPF) serves as reference data and as initial condition (IC) for the first set-up. The IC for the second set-up are composed out of the analytical Sexl–Womersley (SW) velocity profile superimposed with an optimal perturbation (OP). The third set-up is initialised with an unperturbed SW flow and then permanently perturbed using a localised body force (see Section 3.6).

We first tested the effect of the pulsation on spatiotemporal intermittency by performing a DNS initialised with a snapshot of the statistically steady pipe flow (SSPF), as shown in Figure 2a. We refer to these simulations as IC SSPF. Next, we followed Xu et al. [20] and performed a linear non-modal stability analysis to identify the optimal perturbation for the parameter values of interest (Re=2400, Wo=8, and several *A*). This method produces the geometry (radial shape and axial/azimuthal wavenumbers) and the initial time ($t_{0}$) of the perturbation achieving the maximum energy amplification. We used these optimal perturbations on top of the Sexl–Womersley velocity profile as initial conditions for a second set of DNS in order to test whether puffs or helical waves were developed. We refer to these simulations as IC SWOP. In a last step, we performed a third set of DNS with the body force term in Equation (3) to mimic the experimental setup of Xu et al. [17]. All parameter combinations for which we have performed DNS are summarised in Figure 5b.

### 3.1. Temporal Modulation of Statistically Steady Puff Dynamics

Figure 2a shows the typical intermittent behaviour of statistically steady pipe flow at Re=2400. Here, the vorticity is viewed from a reference frame co-moving at the constant bulk speed $u_{s}={\langle u_{b}\rangle }_{t}$. This case was run for 6000 convective time units (Dus) or an equivalent to more than 100 periods beforehand in order to relax from its initial conditions and to let the flow develop its typical patchy and intermittent character: Turbulence is spatially localised and surrounded by laminar regions of relative calm. The time scale of laminar–turbulent interactions is on the order of 100Dus, as can be seen in Figure 5a, where we plot the temporal evolution of the turbulent (volume) fraction (Ft=VturbVpipe) in the computational pipe domain ($V_{\mathrm{pipe}}$). It changes considerably every two or three hundred time units, reflecting the interactions visible in the corresponding space–time diagram. We computed $F_{t}$ based on the streamwise vorticity plotted in Figure 2a and the threshold to separate laminar regions (deep blue in Figure 2) from turbulent ones was set to $\omega_{z}^{2}=4\times 10^{-2}$ in order to match the average turbulent fraction reported by Avila and Hof [12] at Re=2400 (approximately 50%, as in Figure 5b).

We started all pulsatile IC SSPF runs from the same initial flow field and set the initial time to tT=0.25 to match the instantaneous bulk velocity of the pulsation (see Equation (1)) to the one of the steady flow. This ensured a smooth evolution from the initial condition and further allowed us to track the exact same realisations of localised flow structures in space and time as *A* was increased. The resulting spatiotemporal dynamics are shown in Figure 2b–d,f in a frame co-moving at the instantaneous bulk speed

(9) $$\begin{array}{c} x^{*}\left(t\right)=\int_{t_{0}}^{t}u_{b}dt. \end{array}$$

**Figure 6:**
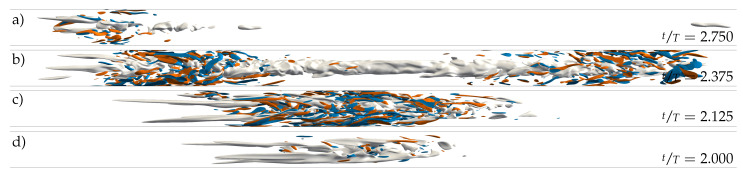
Instantaneous representation of localised turbulent structures in a pulsatile pipe flow (Re=2400, Wo=8, A=0.5). Grey surfaces represent low-speed streaks ($u_{z}^{\prime }=-0.4$$u_{s}$) and blue/red surfaces represent positive/negative axial vorticity (ωz=±8usD). (**a**) Death of downstream puff. (**b**) Splitting event. (**c**) Growing puff. (**d**) Isolated puff. The exact location and time for each snapshot are as indicated in Figure 2d. The direction of the mean bulk flow ($u_{s}$) is always from left to right.

Already at A=0.2, the time scale of the flow modulation (T≈60Dus) dominates the dynamics (Figure 2b). In general, as the amplitude of the pulsation increases, the turbulent fraction in the flow decreases, as seen in Figure 5b. Many structures in the initial flow field decay quickly and do not survive the first acceleration (AC) phase. At A=0.2, only two puffs survive after t=5T, and the dynamics appear to reach an equilibrium state that repeats cyclically. The two surviving puffs grow in intensity and in length during the early deceleration (DC) phase of the flow, and they split into two in the late stages of DC before the minimum flow rate is reached. Out of these two, only the upstream puff survives the entire AC phase and reaches the peak flow rate, where this cycle starts over. Indeed, it is well known that for SSPF, only the upstream puff survives in puff interactions [26]. Overall, it appears that for A=0.2, the flow is clearly self-sustained (above the critical point) and that a successful splitting event may occur at later times. However, the length of the computational domain (100D) may not be sufficient to accommodate three puffs without strong interactions due to the periodic boundary conditions. Similar results were obtained for A=0.4 and 0.5 and are shown in Figure 2c,d; the question of whether, in these cases, the puffs will ultimately decay or successfully split would require substantially longer runs than those performed here and is not further pursued. Figure 6 shows typical localised structures at four equispaced points of the cycle for A=0.5, illustrating the cyclically occurring splitting attempts. In agreement with Xu et al. [15], Xu and Avila [16], these figures show that the surviving puffs (Figure 6d,c) are very similar to the puffs in the steady case (Figure 1c) even at this relatively large pulsation amplitude. For A=0.6, no turbulent structure survived the first pulsation period, and the flow fully relaminarised. We checked amplitudes up to A=1.4 (see Figure 5b). In general, with increasing amplitude, the downstream puff separates farther away from the upstream puff during AC before it dies at almost the end of AC.

### 3.2. Optimal Infinitesimal Perturbations of Pulsatile Pipe Flow

We performed a linear non-modal stability analysis of Sexl–Womersley flow at (Re=2400, Wo=8) and amplitudes up to A=1.6, as described in Section 2.3. For A≤0.4, the optimal perturbation is the same as for statistically steady pipe flow: an axial two-roll configuration (not shown here). For A≥0.5, the optimal perturbation is a streamwise helix and the optimal initial time of perturbation is t0T∈[0.2,0.3]. In Figure 3b,c, we show the optimal perturbation for A=1 at the optimal time of perturbation ($t_{0}$) and at the point of maximum energy amplification ($t_{f}$), respectively. Initially, the optimal helical perturbation is localised very close to the pipe wall at the border of the Stokes layer (δ=12Wo), and it is tilted towards it. Within the rest of the DC phase and the first stages of AC, the perturbation rapidly grows by four orders of magnitude in energy within only 40% of the period. By the time of maximum energy amplification (early AC phase at tT=0.6), the optimal helix has separated from the Stokes layer and moved completely to the outer bulk region, where the stabilising effect of acceleration arrives later. This wall-normal phase lag increases with Wo [13] and can be nicely seen for the profiles close around the peak flow rate (tT=0.5 and 1.0) in Figure 3a. At the end of the process, the helix has been tilted opposite to its original configuration in a process reminiscent of the Orr mechanism. See Xu et al. [20] for more details and for a comprehensive parametric exploration.

### 3.3. Nonlinear Dynamics of Helical Perturbations

In our second set of DNS (IC SWOP), we superimposed the optimal helical perturbation scaled to a small amplitude ($4\times 10^{-2}$ $u_{s}$) on top of the SW profile. All simulations were started at the optimal initial time of perturbation ($t_{0}$). We used a global, as well as an axially localised, helix as initial perturbation and we varied the pulsation amplitude *A* whilst keeping Re=2400 and Wo=8 fixed. In all runs, the global helix exhibited rapid growth, followed by a breakdown into turbulence and immediate decay within the first period, in good agreement with the DNS of Xu et al. [17], for A=0.85, Wo=5.6, and a shorter pipe domain. Our results hence extend their findings to larger *A* and Wo, and are not explicitly shown here.

**Figure 7:**
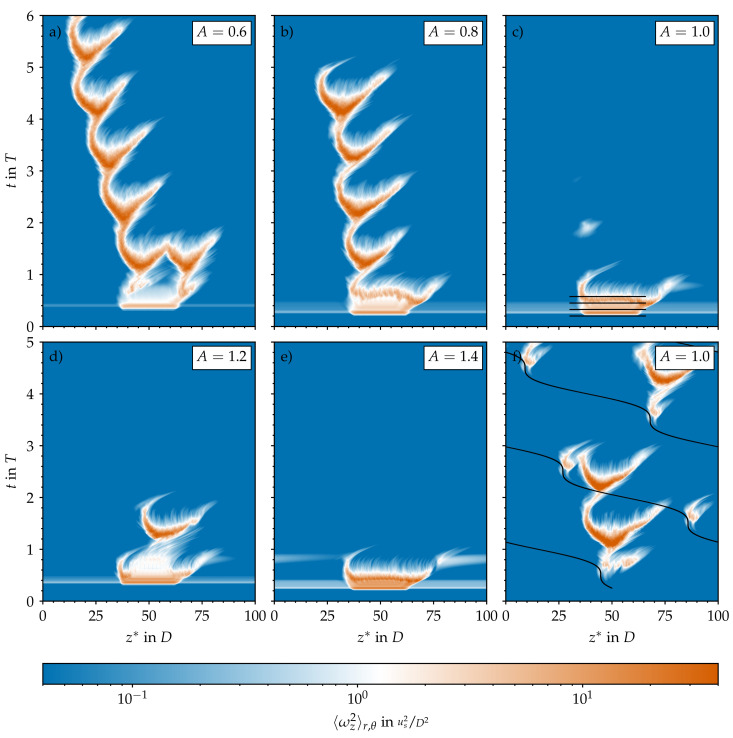
Spatiotemporal representation of the turbulence activity in the pipe domain based on the cross-sectional average of the streamwise vorticity ($\omega_{z}$) plotted on a logarithmic scale and in a co-moving reference frame ($z^{*}$). For pulsatile pipe flow at (Re=2400, Wo=8). (**a**–**e**) For different pulsation amplitudes *A*, always using the SWOP initial condition. Note that the optimal time of perturbation slightly changes with *A*. The horizontal straight lines mark regions for which three-dimensional representations of the localised flow structures are shown in Figure 8. (**f**) For a permanent body force and the unperturbed SW velocity profile as initial condition. The curved black line represents the fixed location of the highly localised body force viewed from the co-moving reference frame. The direction of the mean bulk flow ($u_{s}$) is always from left to right.

**Figure 8:**
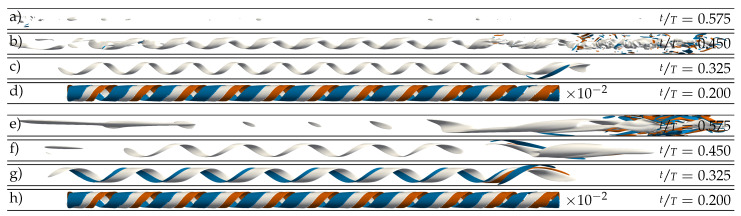
Instantaneous representation of localised turbulent structures in a pulsatile pipe flow DNS at Re=2400, Wo=8, and two different amplitudes. (**a**–**d**) Growth and decay of an initial helix at A=1.0. (**e**–**h**) Development of a puff at A=0.5. Both DNS were initialised at tT=0.2 using the SWOP initial condition. Grey surfaces represent low-speed streaks ($u_{z}^{\prime }=-0.4$$u_{s}$) and blue/red surfaces represent positive/negative axial vorticity (ωz=±8usD). The exact location for each snapshot is as indicated in Figure 2e and Figure 7c, respectively. (**a**) Decay. (**b**) Breakdown into turbulence. (**c**) Amplification of helix. (**d**) Localised optimal helix perturbation. (**e**,**f**) Birth of a downstream puff. (**g**) Amplification of helix. (**h**) Localised optimal helix perturbation. Note that the initial perturbation is two orders of magnitude smaller. The direction of the mean bulk flow ($u_{s}$) is always from left to right.

Using a localised helix as initial condition instead also led to a very similar fate for the helix, but only for A≥0.8 (see Figure 5b). The amplification of the local helix and its subsequent death is shown in Figure 7c–e and Figure 8a–d. For smaller amplitudes (A≤0.8), intermittent puff turbulence emerged after the growth and decay of the initial helix and then was sustained for many periods (see Figure 5). The dynamics of the generated localised puffs are the same as described in Section 3.1 and are exemplarily shown in Figure 8e–h. The puffs that survive AC grow during early DC and attempt to split into two puffs during late DC. In the subsequent AC, the splitting downstream puff decays and leaves only the upstream puff behind to start the cycle over. Figure 2e and Figure 7a,b compare this cycle and its initialisation phase for different amplitudes. For A=0.5, a self-sustaining puff develops only from the downstream end of the amplified helix. For A=0.6, puffs develop from both ends of the localised helix. Shortly thereafter, both puffs interact, which leads to the death of the downstream puff (similar to what happens, for example, in Figure 2b). For A=0.8, a puff develops only from the upstream end of the amplified helix. For this case, the puff is able to survive for four periods before the flow completely relaminarises.

For both large and small amplitudes, the initial optimum perturbation energy is amplified by about two orders of magnitude (Figure 8), which is much less than in the linear case. It is worth noting that perturbations obtained with a non-linear non-modal stability analysis should yield a more effective growth [27]. These methods would help to avoid the discrepancy between linear and non-linear behaviour of perturbations at least before their complete saturation, and should be considered in future works. Our linear optimum perturbation, once introduced into the DNS, also moves towards the bulk region of the pipe; however, before it can complete the growth predicted in the linear analysis, it breaks up into turbulent spots arising at its upstream and downstream ends. For the larger amplitudes, the helix further narrows and develops a turbulent puff with a central low-speed streak before decaying. For the lower amplitudes, on the other hand, the helix opens up again and develops a turbulent spot with several low-speed streaks closer to the wall.

We used a hyperbolic tangent, as in Equation (7), to localise the radial and axial velocities of the helix perturbation in the *z* direction with the parameters Mz=20D and $L_{z}=5D$. The azimuthal velocity was calculated to preserve the divergence-free condition. For a perturbation magnitude of $4\times 10^{-2}$ $u_{s}$, this procedure leaves a remainder of the helix in the rest of the domain, which is everywhere $<10^{-7}$$u_{s}$. This remainder grows dramatically and results in the white bands visible in Figure 2e and Figure 7a–e.

### 3.4. Puff Recovery Length

**Figure 9:**
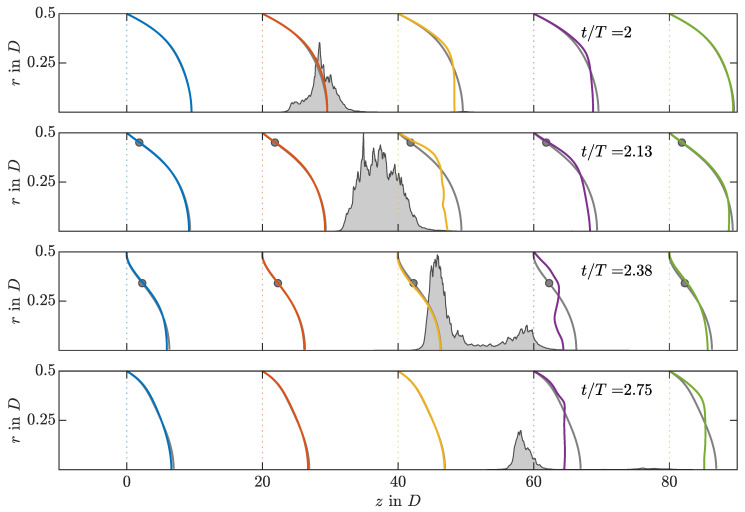
Instantaneous streamwise velocity profiles ($u_{z}$) at five axial locations along the pipe for an IC SWOP simulation at Re=2400, Wo=8, and A=0.5. To not interfere with one another, they are scaled in arbitrary physical units, since, in this representation, only the development in time and deviation from the SW profile are of interest. Thus, the velocity is scaled so its all-time maximum $u_{z}\left(r,\theta =0,z\right)$ is equal to 10D. Each profile is compared with the corresponding instantaneous SW profile (grey lines, also scaled) and its inflection point (grey circles) if they fulfil the Fjortoft criterion. The shaded grey area shows the instantaneous cross-sectional average of the streamwise vorticity (${\langle \omega_{z}^{2}\rangle }_{r,\theta }$) scaled so its all-time maximum is equal to 0.5D.

As has been observed in both strategies, whenever a puff tries to split, only the upstream puff survives. This is a feature common to SSPF and is related to the so-called puff recovery length, which represents the influence length the puff has downstream from its position [26,28]. Its effect can be seen in Figure 9, where the instantaneous $u_{z}$ profile is presented at five axial positions and four time instants for an IC SWOP simulation at Re=2400, Wo=8, and A=0.5. The position of the turbulent puff is presented as a shaded area in terms of axial vorticity. For all phases, the flow quickly recovers the laminar SW profile upstream of the puff location. Downstream of the puff, on the other hand, the flow needs a much longer buffer length to do so. Note the periodic boundary conditions used in our DNS.

### 3.5. Intermittent Production and Dissipation

In order to investigate the physical mechanisms by which puffs arise and survive in pulsatile pipe flow, we computed the production and dissipation of turbulent kinetic energy,

(10) $$\begin{array}{c} P_{\alpha }\;\left(r\right)=-{\langle u_{r}^{\prime }u_{z}^{\prime }\rangle }_{\alpha }\frac{\partial \;{\langle u_{z}\rangle }_{\alpha }}{\partial r}\;\mathrm{and}\;D_{\alpha }\;\left(r\right)=-\frac{1}{R\;e}{\langle \nabla u^{\prime }:\nabla u^{\prime }\rangle }_{\alpha }. \end{array}$$

**Figure 10:**
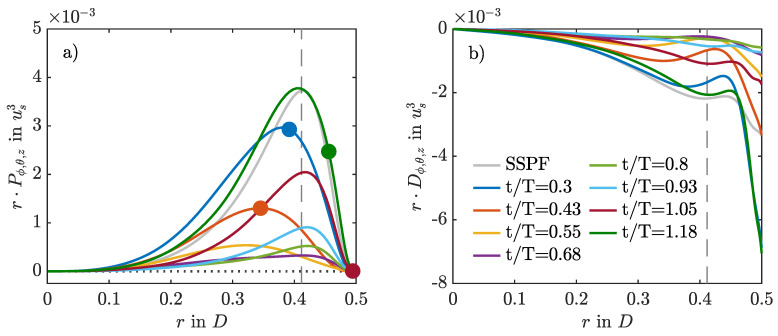
Production (**a**) and dissipation (**b**) of turbulent kinetic energy compared for different phases of the pulsation period for A=0.6 using the SWOP initial conditions. Averages are taken over space- and phase-logged time instants (α=θ,z,ϕ) over four periods of puff dynamics, excluding the initial period without puffs. Circles denote the existence and wall-normal location of the inflection points of the corresponding mean profile $\partial^{2}{\langle u_{z}\rangle }_{\phi ,\theta ,z}/\partial^{2}r=0$ that satisfy the Fjortoft criterion. The vertical dashed line denotes the Stokes layer.

Angled brackets denote averaging with respect to α and prime denotes the fluctuation around the respective average. Here, α can be any combination of averaging in the two homogeneous directions θ and *z*, as well as time *t*, or at a fixed phase ϕ. For the cases where puffs survive, $P_{\theta ,z,\phi }$ and $D_{\theta ,z,\phi }$ are strongly modulated by the pulsation of the flow, as exemplified in Figure 10 for A=0.6. During AC, production and dissipation are low, whereas during DC, they are high. Peak production takes place during the early DC and is very similar to steady pipe flow in terms of magnitude and wall-normal distribution. However, at the phase of maximum production, the dissipation inside the Stokes layer is much more intense than in the steady case. Right after the peak in flow rate, the mean velocity profile develops an inflection point at the wall, which satisfies the Fjortoft criterion [6]. With ongoing deceleration, the inflection point moves away from the wall and catches up with the point of peak production. Both travel together further towards the pipe centre. Near to the minimum flow rate, the unstable inflection point loses the Fjortoft condition (because of new inflection points arising in the velocity profile,) and the production collapses. Hence, it appears that the puff is taking advantage of this inflection point during DC to survive the upcoming AC.

**Figure 11:**
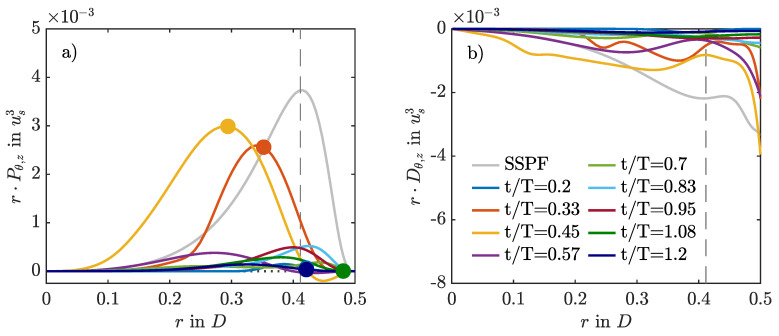
Production (**a**) and dissipation (**b**) of turbulent kinetic energy compared for different phases of the initial pulsation period for A=1 using the SWOP initial conditions.

Figure 11 compares the production and dissipation profiles for the growth and decay of the localised helix during the first pulsation period for A=1. Here, phase-logged time averaging is not possible, and averaging was performed only in the θ and *z* directions. During DC, the rate of production is negative in a small region inside the Stokes layer, meaning that turbulent kinetic energy is fed back to the mean flow and acts as an additional energy sink. This promotes relaminarisation and explains why the helix does not evolve into puff dynamics, as in the low-amplitude cases. Overall, the phenomenology is similar to that reported for oscillatory pipe flow, where negative production causes turbulence decay in cases initialised with fully developed turbulent flow fields of SSPF at high Reynolds numbers [29].

### 3.6. Effect of Local Geometric Imperfections

**Figure 12:**
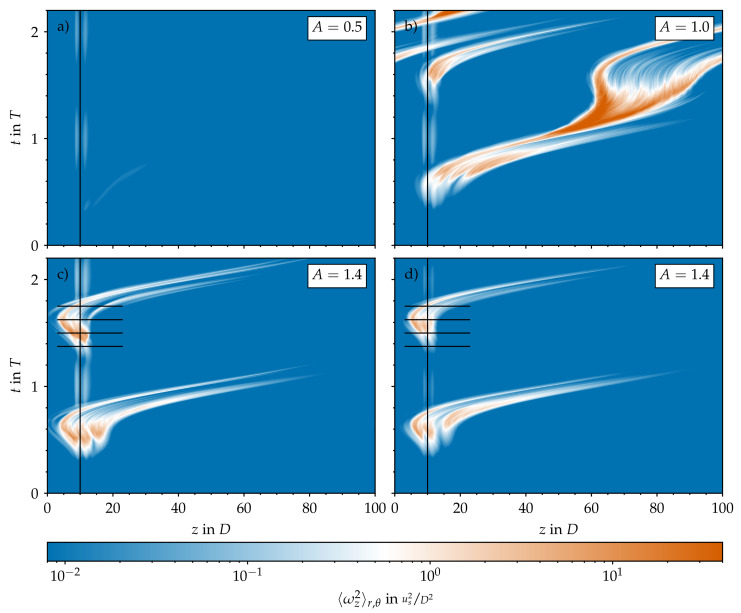
Spatiotemporal representation of the turbulence activity in the pipe domain based on the cross-sectional average of the streamwise vorticity ($\omega_{z}$) plotted on a logarithmic scale and in a stationary reference frame. Pulsatile pipe flow at Re=2400, Wo=8, and different amplitudes *A*. Initial conditions are based on the Sexl–Womersley velocity profile, and there is a permanent body force. (**a**–**c**) Local bump. (**d**) Tilted bump.

We performed a third set of DNS using the laminar SW velocity profile as the initial condition and the volume force described in Section 2.4. For this third set of simulations, we considered only the four amplitudes A∈{0,0.5,1.0,1.4}. In line with the experiments of Xu et al. [17], we found no transition to turbulence at all for the axisymmetric contraction in all cases considered. By contrast, when the force is localised in all three dimensions, the response of the flow depends strongly on the amplitude of the pulsation. For A=0, i.e., statistically steady pipe flow, there is no surge of turbulence or localised transition arising from the bump. This confirms that our force represents a small perturbation to the flow. As we increase the amplitude to A=0.5, some vorticity is generated during peak flow rate, but no turbulent dynamics develop (see Figure 12a). The picture changes for amplitude A=1. As shown in Figure 12b, during early DC, the presence of the bump is able to trigger turbulence in every period. Turbulence grows until late DC, and then laminarises. Occasionally, puffs emerge and are able to survive for more than one period if they interact (again) with the local bump due to the periodic boundary conditions used in our DNS. However, if the upstream puff interacts with a new turbulent spot arising from the bump, both die. For A=1.4, on the other hand, no puffs develop, and the dynamics are solely characterised by bursts of turbulence arising at the bump, which proceed downstream as they decay (see Figure 12c). In all cases, the time at which the perturbation is triggered and grows is in agreement with our non-modal stability analysis and with the experiments of Xu et al. [17].

**Figure 13:**
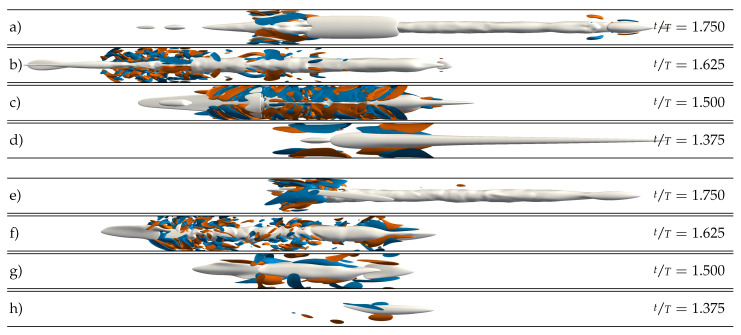
Instantaneous representation of localised turbulent structures in a pulsatile pipe flow DNS at (Re=2400, Wo=8, A=1.4). The DNS was initialised at tT=0.25 using the corresponding SW profile and by introducing a local bump like body force, as described by Equation (3) and Table 1. Grey surfaces represent low-speed streaks ($u_{z}^{\prime }=-0.2$ $u_{s}$) and blue/red surfaces represent positive/negative axial vorticity (ωz=±2usD for all panels except (**d**) and (**h**). There, it is ±0.8usD. (**a**–**d**) Local bump. (**e**–**h**) Tilted bump. The exact instants in time are given in Figure 12c,d. The direction of the mean bulk flow ($u_{s}$) is always from left to right.

Interestingly, the structures that the local bump triggers are mirror symmetric and not helical (see Figure 13a–d). They resemble structures resulting from the optimal non-modal disturbances in pulsatile pipe flow past a constriction [25]. They grow in axial length and magnitude during the late stages of DC while retaining their mirror symmetry. This is only lost in the last stages of DC, as low-velocity streaks form in the centre of the pipe. Finally, either a puff emerges from these streaks, or the flow laminarises during AC.

We also performed simulations with a tilted bump. In this case, the emerging structures exhibit not only mirror-symmetric, but also helical-like features (see Figure 13e–h). They also grow during the late stages of DC and either decay or trigger puffs depending on the pulsation amplitude. From the point of view of spatiotemporal intermittency, their evolution is quite similar to the evolution of the structures triggered by the local bump for all the amplitudes considered, as exemplarily shown in Figure 12c,d.

The fact that different geometric disturbances can trigger different structures is consistent with the non-modal stability analysis of Xu et al. [20]. The analysis showed that the instantaneous Sexl–Womersley profile is linearly unstable (in the quasi-steady limit) during most of the DC phase. Out of all the perturbations that could grow on top of this unstable profile, helical modes have the highest potential to do so. This holds for helical modes spiralling in positive and negative axial directions. That means that, if we were to disturb a flow in a way such that helical modes are excited, unless there is a preferred direction, helical modes and their swirling counterparts can grow simultaneously on top of the laminar flow profile. Our local bump represents a highly symmetric perturbation that allows this to happen, which explains why we observe mirror-symmetric structures. If we introduce some non-symmetric perturbation instead, then we see a preferred direction for the structures to swirl, as in the simulations with the tilted bump.

## 4. Discussion and Conclusions

In agreement with the experiments and simulations of Xu et al. [17], our results show that helical perturbations are able to trigger turbulence in pulsatile pipe flow, but not to maintain it. The helix perturbation grows from the instantaneous linear instability of the laminar flow profile during deceleration. However, during acceleration, the mean profile, which is close to the corresponding SW profile, is linearly stable. Without the unstable character of the profile, the perturbation no longer has its main mechanism to produce turbulent kinetic energy available, and it either completely decays or switches to puff mechanisms to survive. In either case, no helical perturbation is triggered again in the next deceleration phase.

This trend is further confirmed by the simulations that included a body force. For perturbations that seek to mimic the effect of geometric imperfections, and A≥1, turbulence is triggered intermittently every DC and dies during AC, as in the experiments. Thus, for pulsatile pipe flows that are not constantly disturbed, it is the presence of a self-generating puff mechanism (i.e., streak–vortex interaction with lift-up) that guarantees that the flow remains intermittently turbulent throughout many periods.

For puffs to survive in pulsatile pipe flow, plug-like mean profiles must be avoided, as also happens in statistically steady pipe flow [12,26,28,30]. This means that high amplitudes and/or Womersley numbers are detrimental for puffs’ survival, but so are flows with a high fraction of turbulence. This includes cases initialised with a fully turbulent flow field and cases initialised with a helix perturbation in the whole domain. The former has also been shown by Feldmann [29] in purely oscillatory pipe flow at much higher Reynolds numbers. In agreement with Xu et al. [17], global helical perturbations with an initial magnitude of only $4\times 10^{-2}u_{s}$ grow quickly and break up, and turbulence spreads throughout the whole pipe. The resulting highly disturbed flow, whose mean is far from the corresponding SW profile, does not allow puffs to grow. For the helical instability to be able to trigger puffs, it must be localised and surrounded by a laminar flow.

Once they have been successfully triggered, puffs take advantage of two mechanisms: the lift-up mechanism, as in SSPF, and the linear instability of the SW-like profile close to it. The former plays a leading role during late acceleration and early deceleration phases for amplitudes that result in a not-so-plug-like mean profile. The latter has a higher importance for most of the deceleration, where it compensates for the milder gradients of the instantaneous SW-like profile with production of kinetic energy due to its linear instability. The presence of puffs and their corresponding recovery length, in addition to a more intense acceleration phase, make turbulence more intermittent as the amplitude increases.

In future works, a different parametric space will be explored, and the combined effects of body force and random noises will be studied. In addition, physiological-like waveforms with longer deceleration phases will be considered, where the helical instability may have a longer time span to grow.

## Figures and Tables

**Table 1:** Parameters to control the body force term in Equation (3) to model the effect of geometric perturbations: Magnitude ($A_{p}$) and slope (*M*), size (*L*), and location in the radial (*r*), azimuthal (θ), and axial (*z*) direction. Geometric representations of the perturbations are shown in Figure 4.

	$A_{p}$	$M_{z}$ **in** 1D	$L_{z}$ **in** ***D***	$z_{0}$ **in** ***D***	$M_{r}$ in 1D	$r_{0}$ **in** ***D***	$M_{\theta }$	$L_{\theta }$	Δθ
Contraction	0.25	4	2.5	10	100	0.45	20	≥1	0
Bump	0.25	4	2.5	10	100	0.45	20	0.25	0
Tilted Bump	0.25	4	2.5	10	100	0.45	20	0.0625	0.1

## Data Availability

The data we have generated and analysed in this study will be made publicly available soon at https://pangaea.de/.

## Abbreviations

   The following abbreviations are used in this manuscript:

AC	Acceleration
DC	Deceleration
SW	Sexl–Womersley
NSE	Navier–Stokes equations
TGA	Transient growth analysis
DNS	Direct numerical simulation
SSPF	Statistically steady pipe flow
IC SSPF	Cases with a SSPF initial condition
IC SWOP	Cases with a SW profile and optimum perturbation initial condition
